# Spatial–Temporal Correlations between Soil pH and NPP of Grassland Ecosystems in the Yellow River Source Area, China

**DOI:** 10.3390/ijerph19148852

**Published:** 2022-07-21

**Authors:** Xiaoning Zhang, Lili Nian, Xingyu Liu, Xiaodan Li, Samuel Adingo, Xuelu Liu, Quanxi Wang, Yingbo Yang, Miaomiao Zhang, Caihong Hui, Wenting Yu, Xinyu Zhang, Wenjun Ma, Yaoquan Zhang

**Affiliations:** 1 College of Forestry, Gansu Agricultural University, Lanzhou 730070, China; zxn893707607@163.com (X.Z.); nll18893814845@163.com (L.N.); lxy1061062022@163.com (X.L.); lixd@gsau.edu.cn (X.L.); samueladingo557@gmail.com (S.A.); mwj2022781744@163.com (W.M.); zhangyqgs@163.com (Y.Z.); 2 College of Resources and Environmental Sciences, Gansu Agricultural University, Lanzhou 730070, China; yangyb@gsau.edu.cn (Y.Y.); ch19893407596@163.com (C.H.); yuwengting2022@163.com (W.Y.); zxy18793830677@163.com (X.Z.); 3 College of Management, Gansu Agricultural University, Lanzhou 730070, China; m2028578021@163.com; 4 College of Humanities and Law, Northeastern University, Shenyang 110169, China; wquanxi20@163.com

**Keywords:** MODIS, time-lag effect, coupling relationship, contribution index, alpine area

## Abstract

In recent years, ecological concerns such as vegetation destruction, permafrost deterioration, and river drying have been paid much more attention to on the Yellow River Basin in China. Soil pH is regarded to be the fundamental variable among soil properties for vegetation growth, while net primary productivity (NPP) is also an essential indicator to reflect the healthy growth of vegetation. Due to the limitation of on-site samples, the spatial–temporal variations in soil pH and NPP, as well as their intrinsic mechanisms, remain unknown, especially in the Yellow River source area, China. Therefore, it is imperative to investigate the coupling relationship between soil pH and NPP of the area. The study coupled MODIS reflectance data (MOD09A1) with on-site soil pH to estimate spatial–temporal variations in soil pH, explore the response of NPP to soil pH, and assess the extent to which they contribute to grassland ecosystems, thus helping to fill knowledge gaps. Results indicated that the surface spectral reflectance for seven bands could express the geographic pattern of soil pH by applying a multiple linear regression equation; NPP exhibited an increasing trend while soil pH was the contrary in summer from 2000 to 2021. In summer, NPP was negatively correlated with soil pH and there was a lag effect in the response of NPP to soil pH, revealing a correlation between temperate steppes > montane meadows > alpine meadows > swamps in different grassland ecosystems. In addition, contribution indices for temperate steppes and montane meadows were positive whereas they were negative for swamps and alpine meadows, which are apparent findings. The contribution index of montane and alpine meadows was greater than that of temperate steppes and swamps. The approach of the study can enable managers to easily identify and rehabilitate alkaline soil and provides an important reference and practical value for ecological restoration and sustainable development of grassland ecosystems in alpine regions.

## 1. Introduction

Vegetation plays an important role in the exchange of carbon, water, and energy between the soil and the atmosphere through surface albedo, roughness, and evapotranspiration [1]. Vegetation properties are influenced by a variety of environmental factors [2], especially soil properties related to climate regulation and adaptation, biodiversity conservation, water filtration, and carbon sequestration [3,4,5]. There are intricate interactions between vegetation and soil properties at different scales [6], which have become a hot topic for ecological research in the context of global change [7,8].

NPP can reflect ecosystem health and vegetation growth status [9,10]. It represents the total amount of carbon sequestered per unit of time and space by the plant community through photosynthesis [11]. Soil pH, which is closely related to the effectiveness of nutrients in the soil, is one of the most important factors and fundamental components of soil [12,13]. Thus, soil pH may have direct or indirect effects on NPP due to the interaction between vegetation and soil, whereas the relationship between them and their potential mechanisms is not well understood.

Grassland ecosystems are a critical component of terrestrial ecosystems [14] and a vital terrestrial carbon pool [15], playing an indispensable role in maintaining climate stability [16]. The Yellow River source area is an important water conservation region in China, as well as a case of the complex lake alpine grassland underlying surface [17]. Therefore, exploring the coupling relationship between NPP and soil pH in the alpine grassland of the Yellow River source area will provide a useful scientific reference for regional vegetation restoration and ecosystem conservation.

At present, scholars have reported the influence of soil pH and vegetation [18,19,20], but these studies are based on sampled soil data and vegetation data. However, this approach has not been able to achieve monitoring at broad spatial scales and long-term time scales, resulting in limited studies of vegetation–soil pH relationships at regional scales. Studies have attempted to spatially predict soil pH with the advent of remote sensing technology [21,22,23], which has mostly been employed in agricultural research, and there are few studies related to grassland ecosystems. To fill a gap left by previous research, this study provides a method to obtain large-scale changes with long time series using remote sensing, which is beneficial to further research on the coupling relationship between soil pH and NPP in grassland ecosystems.

Based on a review of the literature, the study attempted to quantify the interaction between pH and NPP in the grassland ecosystem utilizing remote sensing. The specific goals were as follows: (1) predict the spatial pattern of soil pH at 10 cm depth, (2) characterize the spatial and temporal variation of pH and NPP, (3) investigate the coupling relationship between pH and NPP, and (4) evaluate the role of pH and NPP in different grassland ecosystem types. 

## 2. Materials and Methods

### 2.1. Study Area

**Figure 1:**
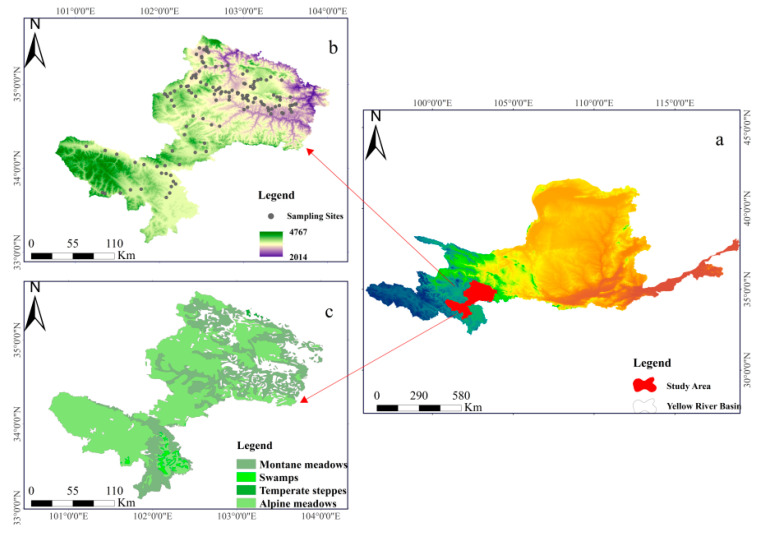
Geographical map of the study area. (**a**) the location of the study area, (**b**) the distribution of sampling points, and (**c**) the four main types of grasslands.

The study was conducted in the Gannan Water Conservation Area, located in the Yellow River source area, among the Tibetan Plateau, the Longnan Mountains, and the Loess Plateau of China (Figure 1). The elevation of the Gannan Water Conservation Area ranges from 2014 m to 4767 m above sea level (a.s.l.). A continental plateau climate predominates in the area; the weather is cold and humid with a high altitude and thin air, where the annual mean air temperature varies from 1 to 3 ºC, while the annual mean precipitation ranges from 400 to 800 mm [24]. Soil types include meadow soil, sub-meadow soil, bog soil, etc. [25]. The land cover types are dominated by grassland and forest, with grassland playing a crucial role [24,26]. The 1:1 million grassland resource map of China was obtained from grassland and ecology (http://ecograss.lzu.edu.cn/) (accessed on 23 August 2021) with the calibration, which mainly used the vegetation–habitat classification system of grassland (VHCS) to classify grassland types. Alpine meadows, montane meadows, temperate steppes, and swamps are the four main types of grasslands found in the area (Figure 1c).

### 2.2. Data Acquisitions and Processing

#### 2.2.1. Remote Sensing Data Acquisition and Processing

The Moderate Resolution Imaging Spectroradiometer (MODIS) Terra surface reflectance products (MOD09A1) and MODIS NPP products (MOD17A2) were used in this study with a spatial resolution of 500 m and intervals of 8 days, which can be downloaded from the United States Geological Survey (USGS) (https://lpdaacsvc.cr.usgs.gov/appeears/explore) (accessed on 4 March 2022). The MOD09A1 product provided a systematic correction for atmospheric factors including aerosols, gases, and Rayleigh scattering to estimate the surface spectral reflectance of seven bands (referred as B1∼B7) [27,28]. 

The monthly data sets were constructed by adopting the maximum value composite method (MVC) for the original reflectance and NPP data from 2000 to 2021 to minimize the influence of atmospheric and cloud contamination [29]. The data from June to August were then averaged to synthesize the summer data. A 90 m DEM (SRTMGL3 DEM) was also downloaded from the USGS. The boundaries of the administrative area and the Yellow River basin were downloaded from the Resource and Environmental Science and Data Center (https://www.resdc.cn/) (accessed on 23 April 2022). 

#### 2.2.2. Soil Samples Data Acquisition and Processing

The soil type, grassland ecosystem type, landscape characteristics, etc., were fully considered in the sampling design. As the reflectance of soil properties and vegetation is more significant during the peak growing season, our sampling period was end of July to mid-August, and the distribution of sampling points is shown in Figure 1b. A total of 130 soil samples were collected from the 0–10 cm soil layer. Each sampling point was uniformly numbered, located using GPS, and latitude and longitude coordinates were recorded. Based on the resolution of MODIS data, two sample quadrats were selected to collect biomass and soil samples at a 500 m × 500 m sample point, and the average value of two sample quadrats spaced 100 m apart from each other was used as the observation value for that sample point. Soil pH was estimated by preparing a suspension with a soil/water ratio of 1:5 [30] using an air-dried and 1 mm sieved soil sample and deionized water.

### 2.3. Methods

#### 2.3.1. Regression Analysis

The Extract by Mask tool in ArcGIS 10.5 was used to generate the B1∼B7 and NPP raster images. Then, the Extract Values to Point tool was used to extract the point data of B1∼B7 and NPP from the raster images that correspond with soil pH point data. Outliers were removed from 130 samples and sorted by sampling points from highest to lowest, with 2/3 of the samples selected as the calibration set and 1/3 of the samples selected as the validation set at equal intervals for model building and accuracy verification, respectively. The normal distribution histogram showed the data suitable for regression analysis. To ensure the reliability of the model, the MODIS data used were kept close to the acquisition time of the field data. 

Correlation analysis of the values of the B1∼B7 at the sampling points and the corresponding pH indicated that there was an internal relationship between the pH and the reflectance (Table 1). Curve estimation was carried out using B1∼B7 as the independent variable and pH as the dependent variable of the calibration set [31]. The primary model types for the soil pH are listed in Table 2, the performance of the models was compared and selected based on the coefficient of determination (*R*^2^) and root mean square error (*RMSE*). The coefficient of determination (*R*^2^) was used to evaluate the relationship between the observed and predicted values, while the root mean square error (*RMSE*) measured the degree of inaccuracy of prediction [32]. The formulas for *R^2^* and *RMSE* are as follows [33]:

(1) $$R^{2}=1-\frac{\sum_{i=1}^{n}{\left(V_{pi}-V_{oi}\right)}^{2}}{\sum_{i=1}^{n}{\left(\overset{\text{-}}{V_{oi}}-V_{oi}\right)}^{2}}$$

(2) $$\mathrm{RMSE}=\sqrt{\frac{1}{n}\sum_{i=1}^{n}{\left(V_{oi}-V_{pi}\right)}^{2}}$$

In Equations (1) and (2), *R^2^* is the coefficient of determination, *RMSE* is the root mean square error, i is the number of the sample point, *n* is the total number of sample points, $V_{oi}\;$is the observed value at sample point *i*, $V_{pi}\;$is the predicted value at sample point *i*. 

**Figure 2:**
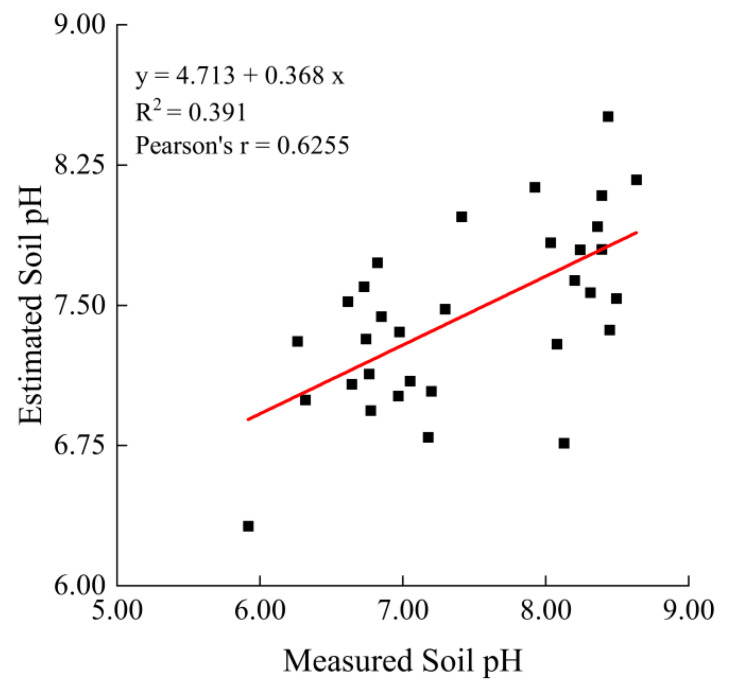
Scatter plots of predicted model accuracy.

B1∼B7 data from the validation set were calculated using multiple linear regression model to derive the predicted soil pH, which was then regressed against the observed soil pH (Figure 2), demonstrating that prediction accuracy was achieved. 

#### 2.3.2. Trend Analysis

In the analysis of the raster data from 2000 to 2021, the least squares method and the F-test based on MATLAB software can be used to obtain the trend and significance test for each grid by iterating image by image. A significant trend was observed when the regression coefficients passed the significance test (F-test, *p* < 0.05) [34].

#### 2.3.3. Cross-Correlation Analysis

In this work, the spatiotemporal coupling between pH and NPP in the summer of 2000–2021 was represented using the Pearson correlation coefficient, and the time-lag correlation was applied to quantify the time-lag effect of pH on NPP [35,36]. Correlation and significance analysis of raster data on the time scale was performed using the MATLAB software package.

#### 2.3.4. Contribution Index

Some researchers have used the contribution index in their research [37,38], which is employed in this paper to measure the degree of contribution of each grassland ecosystem type to pH and NPP. The formula for CI is shown in Equations (3) and (4).

(3) $$CI_{pH}=\left(pH_{i}-pH_{Avg}\right)\times \left(S_{i}/S\right)$$

(4) $$CI_{NPP}=\left(NPP_{i}-NPP_{Avg}\right)\times \left(S_{i}/S\right)$$

In Equation (3), $CI_{pH}$ is the contribution index of grassland ecosystems to *pH*, $pH_{i}$ is the average *pH* of the grassland type *I*, $pH_{Avg}$ is the average *pH* of the entire study area, $S_{i}$ is the area of grassland type I*,* S is the area of the entire study area.

In Equation (4), $CI_{NPP}$ is the contribution index of grassland ecosystems to *NPP*, $NPP_{i}$ is the average *NPP* of the grassland type *i*, $NPP_{Avg}$ is the average *NPP* of the entire study area, $S_{i}$ and S as above.

## 3. Results

### 3.1. Spatial–Temporal Scale Changes in pH and NPP

#### 3.1.1. Temporal Scale Changes in pH and NPP

**Figure 3:**
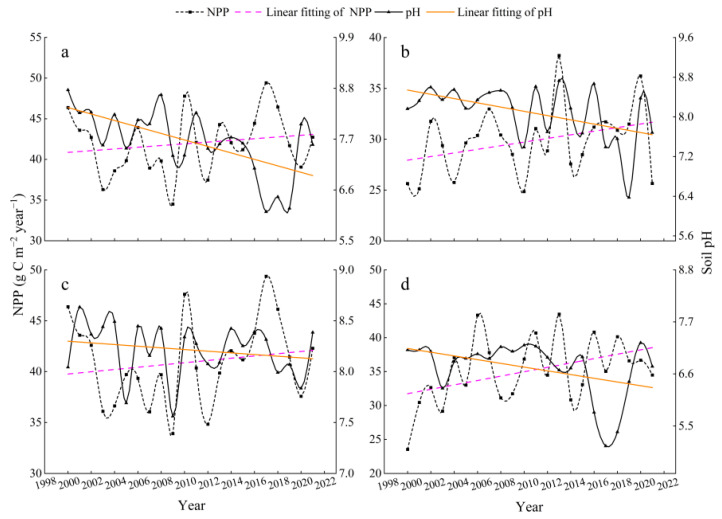
Interannual variations of pH and NPP during 2000–2021: (**a**) pH and NPP in summer, (**b**) pH and NPP in June, (**c**) pH and NPP in July, and (**d**) pH and NPP in August.

In this work, we calculated and analyzed the temporal variations of pH and NPP at monthly, seasonal, and interannual scales using pixels as the minimum unit of calculation. As shown in Figure 3, NPP increases significantly in summer and from June to August (pink dashed line), while pH decreases more rapidly (yellow dashed line). The NPP showed periodic changes with significant fluctuations throughout the summer, with the maximum in 2020 and 2017, and the minimum in 2003 and 2009, respectively. In the different months of the summer, there were variations in the increase in NPP from June to August, with the rate in June and August being higher than that in July; the pH decrease in June and August was significantly higher than that in July, with similar fluctuating trends.

#### 3.1.2. Spatial Scale Changes in pH and NPP

**Figure 4:**
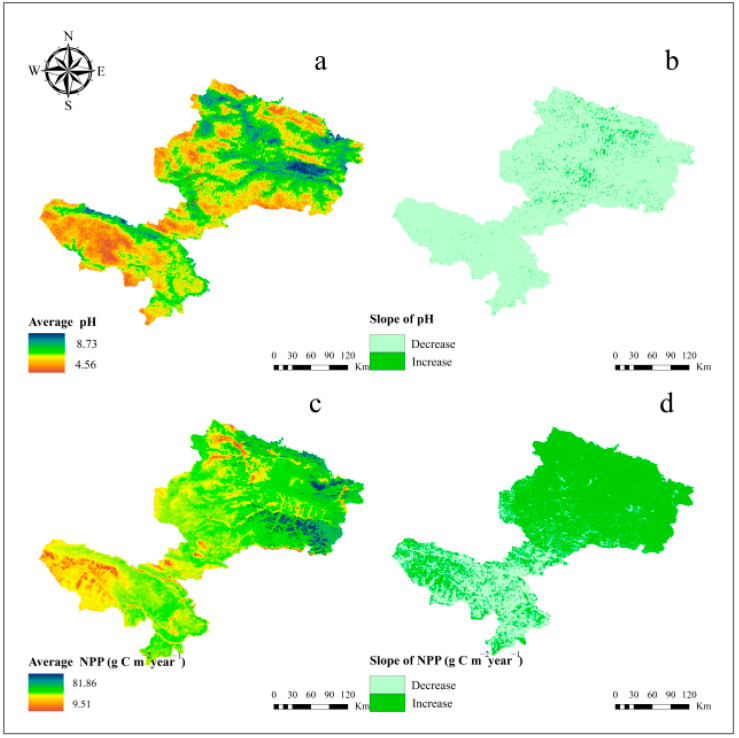
Spatial variation distribution of pH and NPP during 2000–2021: (**a**) the mean pH, (**b**) trend of pH, (**c**) the mean NPP, and (**d**) trend of NPP.

Figure 4 shows the geographic patterns of mean pH and NPP in summer from 2000–2021. Throughout the study area, annual mean pH (Figure 4a) roughly exhibited the distribution characteristics of a high value in the northeast and southwest, while the differences in other regions were on the contrary. The pH trend (Figure 4b) showed an increasing trend in a few northern parts, but a large portion of the territory showed a decreasing trend. The annual mean NPP was higher in the northeast (Figure 4c), indicating that the region has a large carbon sequestration capacity, while the opposite was true in the southwest. Furthermore, the NPP is increasing across the entire northeast region (Figure 4d).

### 3.2. Spatial–Temporal Coupling between pH and NPP

#### 3.2.1. Correlation of pH and NPP

**Figure 5:**
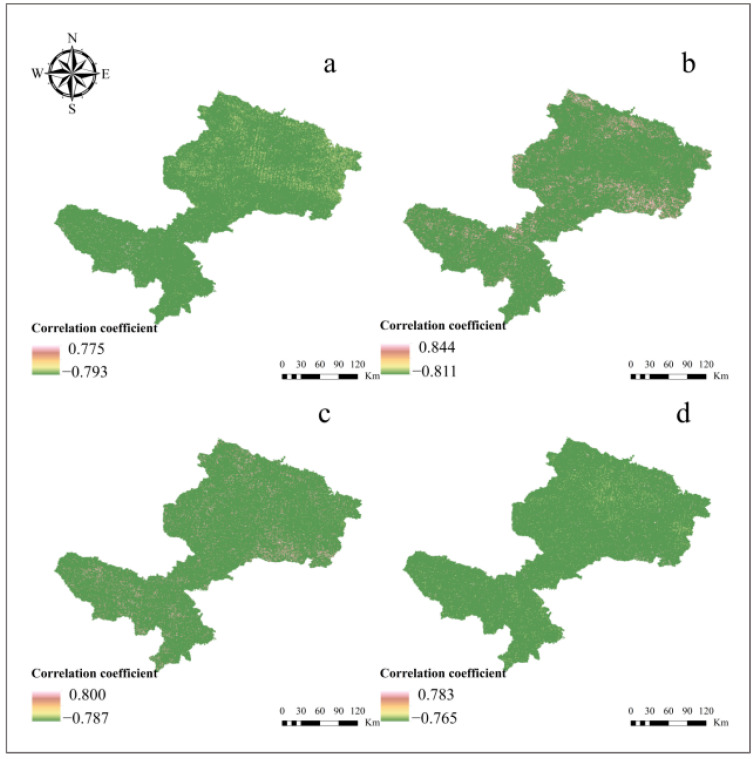
Spatial distribution of the correlation between pH and NPP in summer during 2000–2021: (**a**) the correlation in summer, (**b**) the correlation in June, (**c**) the correlation in July, and (**d**) the correlation in August.

To measure the response of NPP to changes in soil pH, the correlation coefficient between them was calculated spatially based on pixels from 2000 to 2021 (Figure 5). During summer and from June to August, there were negative correlations in most regions, with some regions showing no correlation. The average correlation coefficient for the whole study area was −0.407 (*p* < 0.05) during summer, indicating a moderate negative correlation (Table 3). Additionally, in terms of correlation by month, the strongest correlation was found in June with a correlation coefficient of −0.408 (*p* < 0.05), followed by August with a correlation coefficient of −0.331 (*p* < 0.05), and July with the lowest correlation of −0.317 (*p* < 0.05).

#### 3.2.2. Time-Lag Correlation at the Monthly Scale

**Figure 6:**
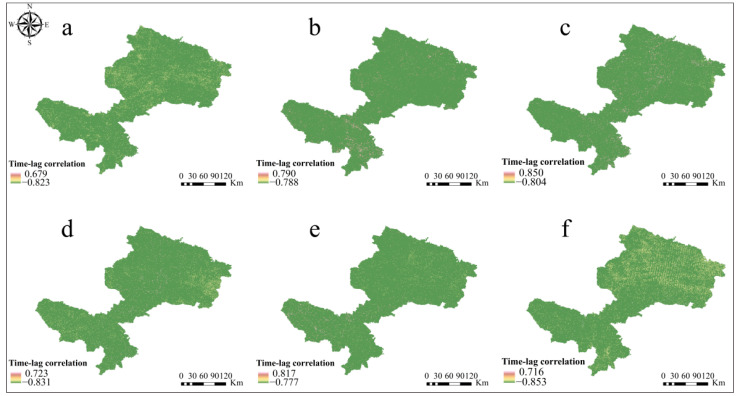
Spatial distribution of the time-lag correlation between pH and NPP in summer during 2000–2021: (**a**) time-lag correlation between pH in June and NPP in July, (**b**) time-lag correlation between pH in June and NPP in August, (**c**) time-lag correlation between pH in July and NPP in August, (**d**) time-lag correlation between pH in July and NPP in June, (**e**) time-lag correlation between pH in August and NPP in June, and (**f**) time-lag correlation between pH in August and NPP in July.

On the monthly scale, there was a negative time-lag correlation between pH and NPP (Figure 6). From the point of view of positive time-lag correlation (Figure 6a–c and Table 4), all of which also showed a negative correlation. The correlation coefficient between pH in June and NPP in July was −0.462 (*p* < 0.05), followed by pH in June and NPP in August, which had a correlation coefficient of −0.242 (*p* < 0.05). The correlation coefficient between pH in July and August in July was weakest at −0.118 (*p* < 0.05). It can also be seen that pH in June and July had a greater effect on NPP in August, with a stronger effect in July.

The negative correlation coefficients with a time lag (Figure 6d–f and Table 4) show that there is a negative correlation between the current monthly NPP and the soil pH in the following month. The correlation coefficients between pH in July and NPP in June or pH in August and NPP in June were −0.317 and −0.203, respectively, and were weakly negative correlations, indicating that NPP in June was responsive to pH in July and August, i.e., the increase in NPP in June would lower pH in July and August. However, pH in August was a moderately negative correlation with NPP in July, which is probably because the study area belongs to the alpine region and reaches the most vigorous vegetation in July and August.

### 3.3. Effects of Grassland Ecosystems on pH and NPP

#### 3.3.1. Temporal Variation of pH and NPP in Grassland Ecosystems

**Figure 7:**
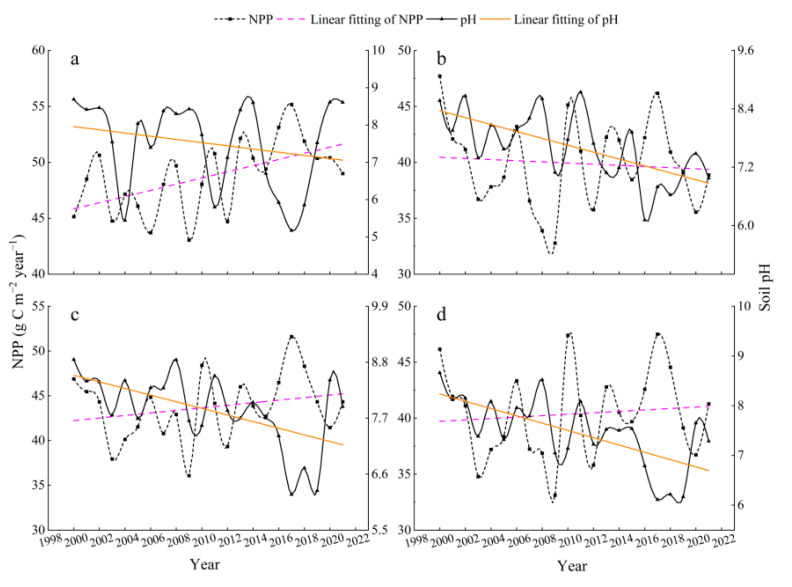
Interannual variations of pH and NPP in grassland ecosystems during 2000–2021: (**a**) pH and NPP of temperate steppes, (**b**) pH and NPP of swamps, (**c**) pH and NPP of montane meadows, and (**d**) pH and NPP of alpine meadows.

Trends in pH and NPP in the four grassland ecosystems have varied over the past 22 years (Figure 7). NPP increased in temperate steppes, montane meadows, and alpine meadows, while it slightly decreased in swamps (pink dashed line). The NPP of temperate steppes had the highest growth rate with a noticeable fluctuation regularity, while montane meadows and alpine meadows were a lower growth rate than temperate steppes. Furthermore, as shown in Figure 7, the trend of pH decreased in four grassland ecosystem types with swamps, montane meadows, and alpine meadows just about right, while temperate steppes were declining at a slower rate than the other types (yellow dotted line). 

#### 3.3.2. Correlation of pH and NPP in Grassland Ecosystems

**Figure 8:**
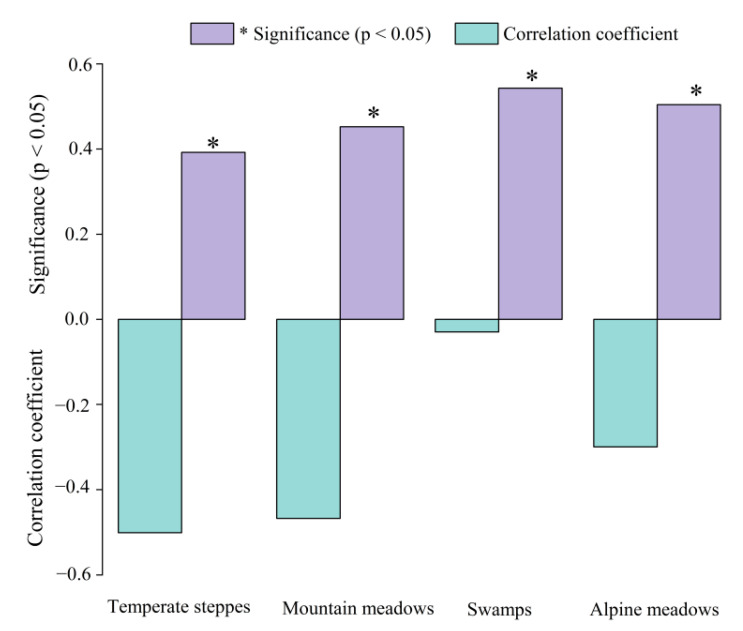
Correlation coefficients of pH and NPP in grassland ecosystems during 2000–2021. * Significant correlation at the 0.05 level (double tail).

To study the effects of pH on NPP in different grassland ecosystem types, the average correlation coefficients of different grassland ecosystems were extracted based on the aforementioned research results (Figure 5). According to the results (Figure 8), the correlation coefficients between pH and NPP for the four grassland ecosystems exhibited negative correlations at seasonal scales. From the perspective of the significance (*p* < 0.05), the correlation of each grassland type was significant. In contrast to the moderate correlation for temperate steppes and montane meadows, followed by the weak correlation between pH and NPP of alpine meadows, and swamps with an extremely weak correlation in summer.

#### 3.3.3. Contribution Index of Grassland Ecosystems to pH and NPP

**Figure 9:**
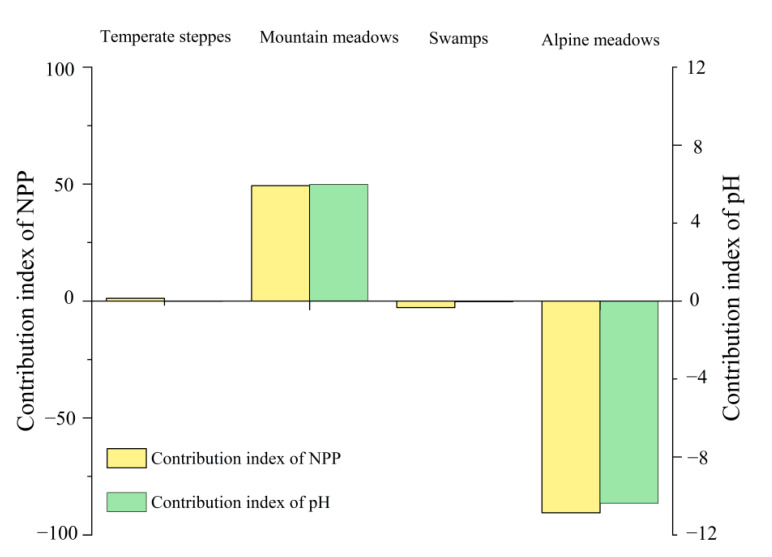
Contribution index of grassland ecosystems to pH and NPP in summer from 2000–2021.

By comparing the contribution indices of each grassland ecosystem type in summer (Figure 9), it could be seen that the contribution indices of pH and NPP were various in all grassland ecosystem types. As shown in (Figure 9), the contribution index of montane meadows and alpine meadows was higher than that of temperate steppes and swamps, as their proportions were 32.70 and 65.53, respectively. In addition, the obvious result was that the contribution indices of NPP for temperate steppes and montane meadows were positive whereas the swamps and alpine meadows were the opposite. In addition, the contribution indices of the pH for montane meadows and alpine meadows played an important role, in which mountain meadows was a negative effect on pH while that of alpine meadows was positive, implying that alpine meadows could lower pH and montane meadows were on the contrary. In terms of absolute value, the great influence of alpine meadows had the greatest impact on pH. Since the contribution index of pH for temperate steppes and swamps was extremely weak, it is not stated here.

## 4. Discussion

### 4.1. Modeling of pH and Its Spatial–Temporal Change

Rising temperatures caused by global warming have degraded the permafrost in the Yellow River source area, having a profound impact on the vegetation and soil conditions [39,40,41]. Because soil pH has a significant impact on plant growth [42], soil microbial changes [43], as well as soil nutrients [44], it has emerged as an important soil variable [23]. More importantly, recent studies have found that soil pH is an important indicator in grassland ecosystem studies [45]. Given the complexity of the ecological environment and the numerous challenges that researchers face when conducting field sampling, the method allowed spatial information to be derived from remote sensing analysis with a large amount of spatially continuous data [46,47].

In previous studies, salinity was estimated using remote sensing techniques [46,48], and these approaches were adopted in estimating pH in this study. The multiple linear regression equation is the superior model presented and it is feasible to estimate soil pH using it over the B1∼B7 (R^2^ = 0.542 and RMSE = 0.473) (Table 2). In other words, the model of soil pH was accurate based on the B1∼B7. The results were promising and showed that soil pH can be reasonably, and easily determined using the B1∼B7 images obtained from Terra surface reflectance products (MOD09A1).

The results of the multiple linear regression analysis between B1∼B7 and soil pH revealed a decreasing trend in pH during the growing season from 2000 to 2014 (Figure 3a). One possible reason was that the national project of returning grazing land to grassland had improved the vegetation cover in the study area since 2000 [49]. Additionally, the apparent decreasing trend in June and August reflected the strongest relationship between pH and NPP (Figure 3b,d), which was expected to lower the pH, implying that B1∼B7 was a better predictor of pH at the beginning and end of the growing season were better predictors of pH. The findings were similar to the research findings of [22] who reported that estimating soil pH at the beginning of the growing season was more effective in spring wheat fields. Furthermore, as indicated in our spatial visualization research (Figure 4a,b) the mean pH was high in the northeastern and southwestern parts of the study region between 2000 and 2021, while it increased the trend in a few northern parts, which may be related to geographic factors (Figure 1b), so further research was needed.

### 4.2. Impact of pH on NPP

With the development of 3S technology, the analysis of the driving forces of NPP in different regions and scales has become more extensive [50]. Temperature, precipitation [51], net radiation [52], and human activities [53,54] have all been shown to influence NPP. In particular, grazing as one of the human activities is a major factor for changes in NPP in grassland ecosystems [55]. Owing to the fact that the vegetation in the study area belongs to alpine grassland ecosystems, the temperature is the main limiting factor for vegetation growth [56,57]. Increasing temperature has alleviated temperature stress on vegetation and created favorable conditions for vegetation growth, which may have contributed to the significant increase in NPP over the past 20 years (Figure 3a and Figure 4d).

In addition, the existing literature using both modeling and experiments has revealed that the effect of soil pH on NPP is quite significant [58], implying that soil properties are also major influencing factors on NPP. Our study of the correlation between soil pH and NPP addressed the problem on a regional scale with the help of remote sensing and filled a gap left by previous studies, which revealed a significant negative correlation between pH and NPP in summer from 2000 to 2021 (Figure 5 and Figure 6). Other findings included that the correlation between pH and NPP was more pronounced in June and August than in July (Figure 5, Table 3), due to the unique climatic characteristics of the study area, including the influence of high elevation and low temperature. In particular, June is the beginning of the growing season for grasslands and August is the end, while July belongs to the high growth season and may influence pH predictions (as described in Section 4.1), which may explain the result of the lower correlation between pH and NPP in July.

Similar to our results, soil pH had distinct effects on community stability in different grassland types [59] and the NPP of each grassland type varies substantially [60]. As illustrated in Figure 7, the decreasing pH in temperate steppes, montane meadows, and alpine meadows was accompanied by an increase in NPP over time, whereas swamps, on the contrary, decreased during 2000–2021. This indirectly implied that pH, as a soil property, was only one factor influencing changes in NPP. An increase in soil temperature [61], which can reduce soil moisture, increase evapotranspiration, and affect plant growth in swamps [62], was cited as the main reason for the change in grassland productivity, whereas temperate steppes, montane grasslands, and alpine grasslands were less susceptible to water stress. This could explain the simultaneous decline in pH and NPP in swamps. Moreover, the negative correlation was validated by the results of the correlation between pH and NPP (Figure 8) and the contribution indices (Figure 9) in various grassland ecosystems. The contribution index of montane and alpine meadows was higher than that of temperate steppes and swamps since the area of each grassland type played a leading role in calculating the contribution index (Figure 9).

### 4.3. Limitations and Prospects

Although remote sensing analysis has become a standard method for monitoring vegetation and soils, the results were still ambiguous due to the complicated spatial–temporal variability of vegetation and soil biochemical processes [63], as well as the effects of resolution of remote sensing data [46]. This study used MODIS products with a moderate spatial resolution of 500 m to quantitatively assess the effects of soil pH on NPP at the time-series scale. The results will provide a scientific basis for the management, restoration, and sustainable development of grassland ecosystems in the Yellow River source area, China. However, as valuable as these methods are, they are still limited by the weak spatial resolution and the limited number of field survey samples.

Our results relate to broader spatial scales, and further studies should employ hyperspectral data for soil properties at smaller regional scales. Meanwhile, in future research, Gaussian process regression, random forest, artificial neural network [64], or kernel ridge regression [23] may be a better attempt to accurately estimate soil properties; data fusion methods should also be adopted to overcome these limitations [65], which would be a better guide for the study of the plant–soil relationship. 

In addition, both NPP and pH vary seasonally [66,67,68,69] yet only summer was investigated, and there are various soil properties while just the pH response to NPP is studied in this paper. As a result, seasonal and monthly variations should be involved in future studies to explain more deeply the intrinsic mechanisms and drivers of coupled relationships in grassland ecosystems. Additionally, last but not least, the results of the study are only representative of our study area and further validation is required to verify that the results for the wider region are consistent with the current results.

## 5. Conclusions

The novelty of this study stemmed from the scarcity of previous research on the effects of soil properties on vegetation using remote sensing. In this work, the gap was bridged by studying the regional variability and spatial correlation of soil pH and NPP. The following results were obtained: (1) From 2000 to 2021, the annual mean pH decreased with increasing NPP in both time and space. (2) Soil pH and NPP showed a moderate negative correlation during the growing season, and their interaction revealed spatial heterogeneity. (3) The correlation between pH and NPP was strongest in the temperate steppe, followed by montane meadows and alpine meadows, with swamps being the weakest in summer. The correlation of each grassland type was significant (*p* < 0.05). (4) The contribution indices of pH and NPP for each grassland ecosystem type were distinct, with the larger contribution indices of montane meadows and alpine meadows implying a more significant contribution for the entire study area. In addition, the evident outcome was that whilst the contribution indices for alpine meadows and swamps were negative, those for temperate steppes and montane meadows were positive. 

The results demonstrated the advantages of remote sensing applications in soil and vegetation research, allowing rapid analysis of their spatial distribution and changes over time. In summary, the results of the technique can be expressed well in topsoil studies, and further research should be conducted to investigate the relationship between soil and vegetation in deeper soils. Furthermore, we should focus on grassland ecosystem types that are sensitive to soil properties and restore areas of poor vegetation cover where possible in future practical work. The cost-effective strategy allows land managers to save time and effort to restore vegetation health and should be widely used in future research.

## Figures and Tables

**Table 1:** Correlation between Terra MODIS bands and soil pH at sampling sites.

Bands	Band1(B1)	Band1(B2)	Band1(B3)	Band1(B4)	Band1(B5)	Band1(B6)	Band1(B7)
Correlation coefficient	0.449 **	−0.356 **	0.406 **	0.241 **	−0.358 **	0.096	0.389 **

** Significant correlation at the 0.01 level (double tail).

**Table 2:** Primary curve estimation of the soil pH.

Model Types	Model Formulas	R^2^	RMSE	F	Sig.
**Multiple linear** **regression model**	**pH = 9.414** ** + ** **35.128B1** ** + ** **20.339B2** ** + ** **17.290B3** ** − ** **63.205B4** ** − ** **26.504B5** ** + ** **13.248B6** ** − ** **4.895B7**	**0.542**	**0.473**	**18.219**	**0.000**
Multiple linear stepwise regression model	pH = 7.815 + 44.391B1 − 43.768B4	0.522	0.542	38.257	0.000
Logarithmic curve model	ln(pH) = 10.915 − 1.240ln(B1)	0.320	0.642	33.439	0.000
Quadratic curve model	pH = 5.550 + 38.938B1 − 131.954B1^2^	0.337	0.638	17.810	0.000
Cubic curve model	pH = 6.507 − 1.839B1 + 385.891B1^2^ − 1962.836B1^3^	0.345	0.639	12.120	0.000

**Table 3:** Correlation of pH and NPP in summer during 2000–2021.

Period	PH	NPP	Correlation Coefficient
Summer	June-August	June-August	−0.407
Current Months	June	June	−0.408
	July	July	−0.317
	August	August	−0.331

**Table 4:** Time-lag correlation of pH and NPP in summer during 2000–2021.

Period	PH	NPP	Correlation Coefficient
Positive time lags	June	July	−0.462
	June	August	−0.242
	July	August	−0.118
Negative time lags	July	June	−0.317
	August	June	−0.203
	August	July	−0.479

## Data Availability

The data that support the findings of this study are available from the corresponding author upon reasonable request.

## References

[1] Pan N., Wang S., Wei F., Shen M., Fu B. (2021). Inconsistent changes in NPP and LAI determined from the parabolic LAI versus NPP relationship. Ecol. Indic..

[2] Lozano-Garcia B., Parras-Alcantara L., Brevik E.C. (2016). Impact of topographic aspect and vegetation (native and reforested areas) on soil organic carbon and nitrogen budgets in Mediterranean natural areas. Sci. Total Environ..

[3] Adhikari K., Hartemink A.E. (2016). Linking soils to ecosystem services—A global review. Geoderma.

[4] Keesstra S.D., Bouma J., Wallinga J., Tittonell P., Smith P., Cerda A., Montanarella L., Quinton J.N., Pachepsky Y., van der Putten W.H. (2016). The significance of soils and soil science towards realization of the United Nations Sustainable Development Goals. Soil.

[5] Bruelheide H., Udelhoven P. (2005). Correspondence of the fine-scale spatial variation in soil chemistry and the herb layer vegetation in beech forests. For. Ecol. Manag..

[6] Liu S., Hou X., Yang M., Cheng F., Coxixo A., Wu X., Zhang Y. (2018). Factors driving the relationships between vegetation and soil properties in the Yellow River Delta, China. Catena.

[7] Sanaei A., Chahouki M.A.Z., Ali A., Jafari M., Azarnivand H. (2018). Abiotic and biotic drivers of aboveground biomass in semi-steppe rangelands. Sci. Total Environ..

[8] Dearborn K.D., Danby R.K. (2017). Aspect and slope influence plant community composition more than elevation across forest-tundra ecotones in subarctic Canada. J. Veg. Sci..

[9] Xue P., Liu H., Zhang M., Gong H., Cao L. (2021). Nonlinear Characteristics of NPP Based on Ensemble Empirical Mode Decomposition from 1982 to 2015—A Case Study of Six Coastal Provinces in Southeast China. Remote Sens..

[10] Li X., Luo Y., Wu J. (2022). Decoupling Relationship between Urbanization and Carbon Sequestration in the Pearl River Delta from 2000 to 2020. Remote Sens..

[11] Liang W., Yang Y., Fan D., Guan H., Zhang T., Long D., Zhou Y., Bai D. (2015). Analysis of spatial and temporal patterns of net primary production and their climate controls in China from 1982 to 2010. Agric. For. Meteorol..

[12] Li Y., Ma J., Xiao C., Li Y. (2020). Effects of climate factors and soil properties on soil nutrients and elemental stoichiometry across the Huang–Huai–Hai River Basin, China. J. Soils Sediments.

[13] Yang J., Guan P., Zhang P., Wu Y., Wang D., Wu D. (2022). Stronger microbial nutrient limitations in subsoil along the precipitation gradient of agroecosystem: Insights from soil enzyme activity and stoichiometry. Soil Discuss..

[14] Liu Y., Yang Y., Wang Q., Du X., Li J., Gang C., Zhou W., Wang Z. (2019). Evaluating the responses of net primary productivity and carbon use efficiency of global grassland to climate variability along an aridity gradient. Sci. Total Environ..

[15] Scurlock J., Hall D. (1998). The global carbon sink: A grassland perspective. Glob. Chang. Biol..

[16] Yan Y., Liu X., Wen Y., Ou J. (2019). Quantitative analysis of the contributions of climatic and human factors to grassland productivity in northern China. Ecol. Indic..

[17] Luo Q., Zhang T., Li Z., Yang J. (2022). Research on the Characteristics of Surface Flux and Surface Parameters of Meadow Underlying Surface in the Source Region of Yellow River. Secur. Commun. Netw..

[18] Jin Z., Luo D., Yu Y., Yang S., Zhang J., Cao G. (2022). Soil pH changes in a small catchment on the Chinese Loess Plateau after long-term vegetation rehabilitation. Ecol. Engl..

[19] Quinto-Mosquera H., Moreno F. (2017). Net primary productivity and edaphic fertility in two pluvial tropical forests in the Chocó biogeographical region of Colombia. PLoS ONE.

[20] Tao D., Chen T., Luo Y., Wu H., Wang X., Wang J., Gao Y. (2021). Belowground net primary productivity and biomass allocation in response to different restoration measures in a salt-alkali-degraded Songnen meadow. Ecol. Indic..

[21] Han Y., Yi D., Ye Y., Guo X., Liu S. (2022). Response of spatiotemporal variability in soil pH and associated influencing factors to land use change in a red soil hilly region in southern China. Catena.

[22] Webb H., Barnes N., Powell S., Jones C. (2021). Does drone remote sensing accurately estimate soil pH in a spring wheat field in southwest Montana?. Precis. Agric..

[23] Zhang Y., Sui B., Shen H., Wang Z. (2018). Estimating temporal changes in soil pH in the black soil region of Northeast China using remote sensing. Comput. Electron. Agric..

[24] Meng B., Gao J., Liang T., Cui X., Ge J., Yin J., Feng Q., Xie H. (2018). Modeling of alpine grassland cover based on unmanned aerial vehicle technology and multi-factor methods: A case study in the east of Tibetan Plateau, China. Remote Sens..

[25] Liu C., Li W., Zhu G., Zhou H., Yan H., Xue P. (2020). Land use/land cover changes and their driving factors in the Northeastern Tibetan Plateau based on Geographical Detectors and Google Earth Engine: A case study in Gannan Prefecture. Remote Sens..

[26] Liu C., Li W., Wang W., Zhou H., Liang T., Hou F., Xu J., Xue P. (2021). Quantitative spatial analysis of vegetation dynamics and potential driving factors in a typical alpine region on the northeastern Tibetan Plateau using the Google Earth Engine. Catena.

[27] An C., Dong Z., Li H., Zhao W., Chen H. (2021). Assessment of Vegetation Phenological Extractions Derived From Three Satellite-Derived Vegetation Indices Based on Different Extraction Algorithms Over the Tibetan Plateau. Front. Environ. Sci..

[28] Liu R. (2016). Compositing the minimum NDVI for MODIS data. IEEE Trans. Geosci. Remote Sens..

[29] Holben B.N. (1986). Characteristics of maximum-value composite images from temporal AVHRR data. Int. J. Remote Sens..

[30] Sharma R., Bella R.W., Wong M.T.F. (2017). Dissolved reactive phosphorus played a limited role in phosphorus transport via runoff, throughflow and leaching on contrasting cropping soils from southwest Australia. Sci. Total Environ..

[31] Wu D., Jia K., Zhang X., Zhang J., El-Hamid A., Hazem T. (2021). Remote sensing inversion for simulation of soil salinization based on hyperspectral data and ground analysis in Yinchuan, China. Nat. Resour. Res..

[32] Ding J., Yang S., Shi Q., Wei Y., Wang F. (2020). Using Apparent Electrical Conductivity as Indicator for Investigating Potential Spatial Variation of Soil Salinity across Seven Oases along Tarim River in Southern Xinjiang, China. Remote Sens..

[33] Adhikari K., Hartemink A.E., Minasny B., Bou Kheir R., Greve M.B., Greve M.H. (2014). Digital mapping of soil organic carbon contents and stocks in Denmark. PLoS ONE.

[34] Yin L., Wang X., Feng X., Fu B., Chen Y. (2020). A Comparison of SSEBop-Model-Based Evapotranspiration with Eight Evapotranspiration Products in the Yellow River Basin, China. Remote Sens..

[35] Chen D., Huang H., Hu M., Dahlgren R.A. (2014). Influence of Lag Effect, Soil Release, And Climate Change on Watershed Anthropogenic Nitrogen Inputs and Riverine Export Dynamics. Environ. Sci. Technol..

[36] Van Meter K.J., Basu N. (2017). Time lags in watershed-scale nutrient transport: An exploration of dominant controls. Environ. Res. Lett..

[37] Yu Z., Yao Y., Yang G., Wang X., Vejre H. (2019). Spatiotemporal patterns and characteristics of remotely sensed region heat islands during the rapid urbanization (1995–2015) of Southern China. Sci. Total Environ..

[38] Sun R., Chen L. (2017). Effects of green space dynamics on urban heat islands: Mitigation and diversification. Ecosyst. Serv..

[39] Cao H., Gao B., Gong T., Wang B. (2021). Analyzing Changes in Frozen Soil in the Source Region of the Yellow River Using the MODIS Land Surface Temperature Products. Remote Sens..

[40] Guo B., Wei C., Yu Y., Liu Y., Li J., Meng C., Cai Y. (2022). The dominant influencing factors of desertification changes in the source region of Yellow River: Climate change or human activity?. Sci. Total Environ..

[41] Ren B., Hu Y., Chen B., Zhang Y., Thiele J., Shi R., Liu M., Bu R. (2018). Soil pH and plant diversity shape soil bacterial community structure in the active layer across the latitudinal gradients in continuous permafrost region of Northeastern China. Sci. Rep..

[42] Yang H., Wu Y., Zhang C., Wu W., Lyu L., Li W. (2022). Growth and physiological characteristics of four blueberry cultivars under different high soil pH treatments. Environ. Exp. Bot..

[43] Liu J., Wang Q., Ku Y., Zhang W., Zhu H., Zhao Z. (2022). Precipitation and soil pH drive the soil microbial spatial patterns in the Robinia pseudoacacia forests at the regional scale. Catena.

[44] Xiong J., Dong L., Lu J., Hu W., Gong H., Xie S., Zhao D., Zhang Y., Wang X., Deng Y. (2022). Variation in plant carbon, nitrogen and phosphorus contents across the drylands of China. Funct. Ecol..

[45] Ma X., Asano M., Tamura K., Zhao R., Nakatsuka H., Wuyunna, Wang T. (2020). Physicochemical properties and micromorphology of degraded alpine meadow soils in the Eastern Qinghai-Tibet Plateau. Catena.

[46] Tran T.V., Tran D.X., Myint S.W., Huang C.-Y., Pham H.V., Luu T.H., Vo T.M. (2019). Examining spatiotemporal salinity dynamics in the Mekong River Delta using Landsat time series imagery and a spatial regression approach. Sci. Total Environ..

[47] Tian M., Yang X., Ran L., Su Y., Li L., Yu R., Hu H., Lu X.X. (2019). Impact of land cover types on riverine CO_2_ outgassing in the Yellow River source region. Water.

[48] Gorji T., Sertel E., Tanik A. (2017). Monitoring soil salinity via remote sensing technology under data scarce conditions: A case study from Turkey. Ecol. Indic..

[49] Shao H., Sun X., Wang H., Zhang X., Xiang Z., Tan R., Chen X., Xian W., Qi J. (2016). A method to the impact assessment of the returning grazing land to grassland project on regional eco-environmental vulnerability. Environ. Impact Assess..

[50] Zhao P., Wang D., He S., Lan H., Chen W., Qi Y. (2020). Driving forces of NPP change in debris flow prone area: A case study of a typical region in SW China. Ecol. Indic..

[51] Wang Z., Zhong J., Lan H., Wang Z., Sha Z. (2019). Association analysis between spatiotemporal variation of net primary productivity and its driving factors in inner mongolia, china during 1994–2013. Ecol. Indic..

[52] Jiao W., Chen Y., Li W., Zhu C., Li Z. (2018). Estimation of net primary productivity and its driving factors in the Ili River Valley, China. J. Arid Land..

[53] Zhang Y., Zhang C., Wang Z., Chen Y., Gang C., An R., Li J. (2016). Vegetation dynamics and its driving forces from climate change and human activities in the Three-River Source Region, China from 1982 to 2012. Sci. Total Environ..

[54] Zhang Y., Hu Q., Zou F. (2021). Spatio-Temporal Changes of Vegetation Net Primary Productivity and Its Driving Factors on the Qinghai-Tibetan Plateau from 2001 to 2017. Remote Sens..

[55] Chi D., Wang H., Li X., Liu H., Li X. (2018). Assessing the effects of grazing on variations of vegetation NPP in the Xilingol Grassland, China, using a grazing pressure index. Ecol. Indic..

[56] Liu H., Mi Z., Lin L., Wang Y., Zhang Z., Zhang F., Wang H., Liu L., Zhu B., Cao G. (2018). Shifting plant species composition in response to climate change stabilizes grassland primary production. Proc. Natl. Acad. Sci. USA.

[57] Wang Y., Xiao J., Ma Y., Luo Y., Hu Z., Li F., Li Y., Gu L., Li Z., Yuan L. (2021). Carbon fluxes and environmental controls across different alpine grassland types on the Tibetan Plateau. Agric. For. Meteorol..

[58] Stevens C.J., Lind E.M., Hautier Y., Harpole W.S., Borer E.T., Hobbie S., Seabloom E.W., Ladwig L., Bakker J.D., Chu C. (2015). Anthropogenic nitrogen deposition predicts local grassland primary production worldwide. Ecology.

[59] Liu K., Liu Z., Zhou N., Shi X., Lock T.R., Kallenbach R.L., Yuan Z. (2022). Diversity-stability relationships in temperate grasslands as a function of soil pH. Land Degrad. Dev..

[60] Miehe G., Schleuss P.-M., Seeber E., Babel W., Biermann T., Braendle M., Chen F., Coners H., Foken T., Gerken T. (2019). The Kobresia pygmaea ecosystem of the Tibetan highlands—Origin, functioning and degradation of the world’s largest pastoral alpine ecosystem Kobresia pastures of Tibet. Sci. Total Environ..

[61] Wu G.-L., Cheng Z., Alatalo J.M., Zhao J., Liu Y. (2021). Climate Warming Consistently Reduces Grassland Ecosystem Productivity. Earths Future.

[62] Vicente-Serrano S.M., Gouveia C., Julio Camarero J., Begueria S., Trigo R., Lopez-Moreno J.I., Azorin-Molina C., Pasho E., Lorenzo-Lacruz J., Revuelto J. (2013). Response of vegetation to drought time-scales across global land biomes. Proc. Natl. Acad. Sci. USA.

[63] Li D., Luo H., Hu T., Shao D., Cui Y., Khan S., Luo Y. (2020). Identification of the Roles of Climate Factors, Engineering Construction, and Agricultural Practices in Vegetation Dynamics in the Lhasa River Basin, Tibetan Plateau. Remote Sens..

[64] Yang H., Hu D., Xu H., Zhong X. (2020). Assessing the spatiotemporal variation of NPP and its response to driving factors in Anhui province, China. Environ. Sci. Pollut. Res..

[65] Yan Y., Liu X., Wang F., Li X., Ou J., Wen Y., Liang X. (2018). Assessing the impacts of urban sprawl on net primary productivity using fusion of Landsat and MODIS data. Sci. Total Environ..

[66] Bao G., Chen J., Chopping M., Bao Y., Bayarsaikhan S., Dorjsuren A., Tuya A., Jirigala B., Qin Z. (2019). Dynamics of net primary productivity on the Mongolian Plateau: Joint regulations of phenology and drought. Int. J. Appl. Earth Obs..

[67] Dong Y., Yang J.-L., Zhao X.-R., Yang S.-H., Mulder J., Dorsch P., Zhang G.-L. (2022). Seasonal dynamics of soil pH and N transformation as affected by N fertilization in subtropical China: An in situ 15N labeling study. Sci. Total Environ..

[68] Guo D., Song X., Hu R., Cai S., Zhu X., Hao Y. (2021). Grassland type-dependent spatiotemporal characteristics of productivity in Inner Mongolia and its response to climate factors. Sci. Total Environ..

[69] Yamashita N., Ohta S., Sase H., Kievuttinon B., Luangjame J., Vsaratana T., Grivait H. (2011). Seasonal changes in multi-scale spatial structure of soil pH and related parameters along a tropical dry evergreen forest slope. Geoderma.

